# Physical activity and sleep during the first week of anorexia nervosa inpatient care

**DOI:** 10.1371/journal.pone.0260077

**Published:** 2021-11-16

**Authors:** Billy Langlet, Fannie Vestermark, Josefin Stolt, Modjtaba Zandian, Per Södersten, Cecilia Bergh

**Affiliations:** 1 Division of Clinical Geriatrics, Center for Alzheimer Research, Department of Neurobiology, Care Sciences and Society, Karolinska Institutet, Stockholm, Sweden; 2 Mandometer Clinic, Stockholm, Sweden; CNRS, University of Strasbourg, FRANCE

## Abstract

**Background:**

Individuals with Anorexia Nervosa are often described as restless, hyperactive and having disturbed sleep. The result reproducibility and generalisability of these results are low due to the use of unreliable methods, different measurement methods and outcome measures. A reliable method to measure both physical activity and sleep is through accelerometry. The main purpose of the study was to quantify the physical activity and sleeping behaviour of anorexia nervosa patients. Another purpose was to increase result reproducibility and generalisability of the study.

**Material and methods:**

Accelerometer data were collected from the first week of treatment of anorexia nervosa at an inpatient ward. Raw data from the Axivity AX3^©^ accelerometer was used with the open-source package GGIR for analysis, in the free statistical software R. Accelerometer measurements were transformed into euclidean norm minus one with negative values rounded to zero (ENMO). Physical activity measurements of interest were 24h average ENMO, daytime average ENMO, inactivity, light activity, moderate activity, and vigorous activity. Sleep parameters of interest were sleep duration, sleep efficiency, awakenings, and wake after sleep onset. The sleep duration of different age groups was compared to recommendations by the National Sleep Foundation using a Fisher’s exact test.

**Results:**

Of 67 patients, due to data quality 58 (93% female) were included in the analysis. Average age of participants was 17.8 (±6.9) years and body mass index was 15.5 (±1.9) kg/m^2^. Daytime average ENMO was 17.4 (±5.1) m*g*. Participants spent 862.6 (±66.2) min per day inactive, 88.4 (±22.6) min with light activities, 25.8 (±16.7) min with moderate activities and 0.5 (±1.8) min with vigorous activities. Participants slept for 461.0 (±68.4) min, waking up 1.45 (±1.25) times per night for 54.6 (±35.8) min, having an average sleep quality of 0.88 (±0.10). 31% of participants met sleep recommendations, with a significantly higher number of 6–13 year old patients failing to reach recommendations compared to 14–25 year old patients.

**Conclusion:**

The patient group spent most of their time inactive at the beginning of treatment. Most patients failed to reach sleep recommendations. The use of raw data and opensource software should ensure result reproducibility, enable comparison across points in treatment and comparison with healthy individuals.

## Introduction

Anorexia Nervosa (AN) was first clinically described in 1873 by English physician William Gull as a disease affecting mostly young women, characterised by a loss of appetite leading to extreme emaciation and amenorrhea [1]. He also noted patients being restless and active, commenting that ‘This was in fact a striking expression of the nervous state, for it seemed hardly possible that a body so wasted could undergo the exercise which seemed agreeable’. Since then, authors have frequently used words such as high levels of physical activity (PA), hyperactivity and similar to characterise behaviours of this patient group [2]. A biological hypothesis for this behaviour is that when energy stores are low the requirement to forage for food is high and when energy stores are high there is less incentive expending energy to acquire food. The hypothesis is strengthened by the increased activity observed in animals when deprived of food [3]. Similar effects have been observed on sleep, where larger food portions result in longer sleep durations and food starvation results in reduced sleep and a more ‘vigilant’ state [4]. These findings have in part been corroborated in humans [5] and could explain why sleep disturbances, and sleep disorders such as insomnia are commonly encountered in AN patients [6, 7].

Most studies measuring PA in AN patients have employed questionnaires and activity diaries [2]. However, these methods are associated with multiple biases (e.g., recall bias) and often provide conflicting results when compared to objective measurements in the same study [8, 9]. Meanwhile, doubly labelled water (DLW), has proven reliable in measuring daily energy expenditure [10], but its high cost result in small samples sizes (n = 6 to 12) [11–13]. Another option to objectively measure PA is through accelerometry, which entails using a device that measures acceleration (in G-force, *g*) across one or more planes. The device is usually placed on the waist, wrist or thigh and can be worn for several consecutive days [14]. A study in 1985, measuring kinetic energy at the wrist and ankle, found positive relationship between activity and weight gain of hospitalised AN patients [15]. A recent systematic review in part corroborate these findings, concluding that involuntary (i.e., subconscious) PA increases with weight gain [2]. However, the authors emphasize the difficulty of interpreting the literature due to the many different definitions of ‘problematic physical activity’ [2]. Meanwhile, studies investigating the difference in PA between AN patients and Healthy Controls (HC) have reached contradicting results, where some studies have found that AN patients are more active than healthy controls [16, 17], others have found there to be no significant difference [8, 18] or that AN patients are less active [19]. Reasons for this discrepancy is likely a combination of the use of different i) devices, ii) algorithms (often proprietary, where the raw data is kept from the researcher), iii) cut-off points for activity levels, iv) outcome measures (counts, time spent on feet, steps, etc.) and v) body placement [8, 16–20]. To complicate matters further, some accelerometer studies have found a larger individual variation in the AN group than the HC group, which may indicate the presence of two distinctly different AN groups [8, 16, 18, 19]. One such study found that, patients with low PA levels increased their activity during therapy, while patients with high PA levels reduced their activity during therapy [21]. Despite there being no consensus in the literature regarding the activity of AN patients, it seems high activity before, during and after treatment can prevent weight gain [22–24]. As such, monitoring PA prior, during and post-treatment could be a useful method to identify high-risk subjects and improve treatment outcomes.

The gold standard to measure sleep is polysomnography (PSG) [25]. PSG studies which compare sleep of AN patients to HC have found AN patients to have longer wake after sleep onset (WASO), more awakenings, lower sleep efficiency [26, 27] and in some cases shorter slow wave [27] or rapid eye movement (REM) sleep [26]. Other studies have failed to corroborate these findings [28, 29]. In a review, researchers conclude that while most eating disorder patients display a normal sleep pattern, there may be a subgroup which suffer from sleep disturbances [30]. Reasons for the discrepancy of polysomnography studies is likely a result of heterogenous samples and low sample sizes. Since polysomnography can be uncomfortable for patients and difficult to employ in real-life and clinical settings an alternative method could be accelerometry, which appears able to provide reliable information on sleep parameters [31]. Only two studies have used accelerometers to measure sleep in patients with AN [32, 33]. One found no significant difference between AN and HC in total sleep time, WASO or sleep efficiency [32]. The second study found that AN patients had significantly lower total sleep time and sleep onset latency compared to HC, which normalised upon weight restoration [33]. A reason for the discrepancy between studies may be that the AN patients of the first study had a higher body mass index (BMI = 16.8), compared to the patients in the second study (BMI = 14.5). Which would suggest the BMI level sufficient to promote disturbed sleep patterns is below 16.8. Some studies suggest patients with sleep disturbances have more severe eating disorder symptoms and worse treatment outcomes [34]. Understanding what constitutes unhealthy sleep patterns at different times of treatment may therefore be an important step towards improving treatment outcomes.

The primary objective was to describe the PA and sleeping behaviour of AN patients during the first week of treatment in an inpatient ward using accelerometers. The secondary objective was to increase result reproducibility, defined as “the production of corroborating results in a new study, having followed the same experimental methods” [35], and generalisability of measurements in the current population by using open-source algorithms and an accelerometer which allows extraction of raw data.

## Materials and methods

### Participants

The study was of an observational nature. Participants consisted of AN patients admitted to inpatient wards at the Mandometer clinics in Stockholm, Sweden. AN diagnosis was carried out by a physician following criteria from the Diagnostic and Statistical Manual of Mental Disorders (DSM-5) [36]. Admittance to inpatient care requires one of the following criteria, a BMI <14, a resting heart rate <46 beats/min, a serum potassium <3.2 mmol/L, a body temperature < 36°C or a risk of suicide. To receive treatment patients had to apply for treatment through self-referral or admittance online or get a physician referral. In this study only measurements initiated within seven days after admission were included. Demographic data on age, sex, weight, height, previous treatments, relapse, onset, and illness duration was collected by a physician upon admission to the inward. Age, sex, weight, height and previous treatments was objectively measured. Illness onset, illness duration and relapse were reported by patients or caregivers.

### Treatment protocol

When treated at the inpatient ward, patients are monitored by clinical staff 24-hours per day. Patients are not allowed to leave the clinic without a doctors approval. However, treatment is voluntary, therefore patients can withdraw from treatment at any time. The cornerstones of treatment are: i) reduction of PA, ii) thermal treatment and rest, iii) normalization of eating behaviour and satiety, and iv) social and psychological reconstruction. Objective activity measurements (e.g., accelerometers and pedometers) have been used in the clinic to reduce PA for approximately 15 years, by providing patients with quantifiable feedback on their behaviour. Previously accelerometers were mainly used for patients who displayed a slower than expected weight increase. However, recently a protocol was established where the first week is measured for all in-patients. Clinicians also supervise patients and provide suggestions on calm activities to reduce PA. Also, patients with a BMI <16 are required to use a wheelchair when traveling longer distances, for example in connection with visits from relatives. At the inpatient ward there are no sports activities or therapeutic elements that involve physical activity. To reduce anxiety before and after meals patients rest in heated rooms (≥40 °C) or under thermal blankets. Eating behaviour is normalized by real-time feedback on eating rate from a medical device (Mandometer^®^). To enable social reconstruction patients are first removed from the environment in which the disorder arose, where stressors maintain their illness, to the inward. Patients are then gradually introduced to the environment again after having received training to better cope with stressors. Patients also work with therapist to set short- and long-term goals related to starting school, contacting peers, understanding the disease, as well as body acceptance and rebuilding relationships with friends and family. In addition to the cornerstones of treatment, to improve sleep habits, patients go to bed at latest 22:00, turn in their devices before bedtime and the clinical staff make rounds at least once an hour during the night to ensure patients are sleeping.

### Physical activity and sleep measurements

To measure physical activity and sleep parameters patients were given a 3-axis accelerometer, (Axivity AX3^©^, Axivity Ltd, United Kingdom) within the first week of being admitted to treatment. Patients were instructed to wear the accelerometer on the non-dominant wrist 24 hours per day, for 7 consecutive days and only take it off during showers. The accelerometer was set at a sampling frequency of 100 Hz. The accelerometer facilitates collection of raw acceleration data that can be processed with multiple software. The original band, which housed the accelerometer, caused skin irritation in some patients, and was therefore replaced with a soft elastic sweatband. To ensure fidelity the same dietician provided patients with the accelerometer along with information on how to use it.

The physical activity variables of interest were 24h average euclidean norm minus one with negative values rounded to zero (ENMO), daytime average ENMO, inactivity, light activity, moderate activity and vigorous activity. 24h average ENMO is the average ENMO between hour 0 to 24 of a day. Daytime average ENMO is the average ENMO between sleep termination and sleep onset of a day. Thresholds were set to 0–39 ENMO = Inactivity, 40–99 = Light activity, 100–399 = Moderate activity and ≥400 ENMO = Vigorous activity.

Sleep variables of interest were sleep duration, sleep efficiency, awakenings, and wake after sleep onset (WASO). Sleep duration was defined as the duration between sleep onset and termination. Sleep efficiency was defined as the proportion of time spent sleeping from onset to termination: (sleep duration–WASO) / sleep duration. Sleep efficiency ranges from 0 to 1, where a score of 1 means the individual did not wake between sleep onset and termination. Awakenings was defined as the number of times a person was awake >5 minutes during the sleep period. WASO was defined as the sum of the time a person was awake between sleep onset and sleep termination (i.e., during the sleep duration period). Sleep onset latency could not be estimated because patients are allowed to go to bed earlier than the required bedtime (22:00).

### Data processing

For patients <20 years, BMI-Z scores were converted into equivalent BMI to enable comparison across ages (age adjusted BMI) [37]. Age adjusted BMI was derived by first calculating z-score for patients below 20 years of age, based on the World Health Organizations (WHO) Child Growth Standards, then converting the z-score back to BMI based on the adult average and variance. Accelerometer raw data were extracted from the accelerometer and processed in R using the GGIR package v. 2.1–0 [38]. The GGIR package converts 3-axis raw acceleration data (m/s^2^) to the metric “euclidean norm minus one with negative values rounded to zero” (ENMO, also known as vector magnitude), which uses the unit milligravity (m*g*) and assumes that earth’s gravity is 1*g*. The calculation is as follows: $\left(\left(\sqrt{{x1}^{2}+{x2}^{2}+{x3}^{2}}\;\right)-1)*1000\right)$, where x1, x2, and x3 are the *g* from each axis. Data is then auto-calibrated according to the local gravity and aggregated to 5s epochs [39]. Non-wear time is detected using 15-minute blocks with a 60-minute sliding window and abnormally high acceleration classified as corrupt data. Sleep was analysed based on the angle variability of the z-axis relative to horizontal plane and the movement of the arm [40]. Results presented in this text were derived from the wrapper function of the GGIR package ‘g.shell.GGIR(mode = 2:5, datadir = <data folder>, outputdir = <output folder>, do.report = c(2,4,5))’, which, sets the light, moderate and vigorous intensity levels to 40, 100 and 400 ENMO respectively [41]. Day and summary results were taken from the 24-hour time-use analysis, rather than from the time of waking up one day to the time of waking up the next. In practice this means data was analysed on a day-to-day basis, from hour 0 to hour 24. These analyses methods follow the UK Biobank study where they were first developed and validated.

### Statistical analysis

Compliance was based on wear time, including only patients who wore the accelerometer ≥16h/day for ≥5 consecutive days in the statistical analysis. To identify if there was an age difference in patients who met sleep recommendations, the sleep duration of different age groups was compared to sleep duration recommendations by the National Sleep Foundation [42], using a Fisher’s exact test. Values are expressed as mean (± standard deviation), unless otherwise specified. The threshold for significance in statistical tests were *p*<0.05. Pearson’s product moment correlation was used to test the association between demographic data (age, BMI, onset, and illness duration) and PA and sleep parameters. The Pearson’s correlation thresholds were set at R; <0.30 (weak), 0.30–0.50 (fair), 0.50–0.70 (good), >0.70 (strong) [43]. For each correlation analysis two tests were run, one with logarithmic transformation and outlier exclusion and one without. To test the normality assumption a Shapiro-Wilk normality test was performed. Average ENMO, daytime average ENMO and moderate activity level parameters failed the Shapiro-Wilk test and were logarithmically transformed. Outliers were defined as observations more than 1.5 times the interquartile range above the upper quartile or more than 1.5 times the interquartile range below the lower quartile. A total of 4, 5 and 6 outliers were removed from age, illness onset and illness duration analyses, respectivelly. Since both correlation tests resulted in correlations values within the same threshold range the non-transformed test with outliers is presented in the Results.

### Ethical permission

The research was approved by the regional Ethical Review Board of Stockholm (Dnr: 2013/1559-31/4). Patients 18 years or older signed a consent form, while younger patients signed an assent form and their legal guardians signed a consent form upon admission to the clinic.

## Results

### Demographic data

Of 67 patients, 3 were excluded due to corrupt data (non-readable files) and 6 due to low compliance (Table 1). Of the remaining 58, 54 (93%) were females. Thirty-one (53%) patients had previously been treated for eating disorders, of which 5 (16%) had been treated to remission. At the beginning of treatment 17 (29%) participants used antidepressants, 6 (10%) used anxiolytics and 17 (29%) used sleeping pills.

**Table 1:** Demographic data of patients.

	Mean	Standard deviation	Range
Age, yrs.	17.8	6.9	10.8–46.8
Height, cm	163.0	7.4	145.5–178.0
Weight, kg	41.2	6.5	28.6–54.4
BMI, kg/m^2^	15.5	1.9	11.0–18.2
Adjusted BMI, kg/m^2^	16.1	2.1	11.0–18.2
Illness onset, yrs.	14.6	3.7	5.0–26.8
Illness duration, yrs.	3.3	5.3	0.0–30.0

Adjusted BMI = the BMI of patients below the age of 20, adjusted to adult BMI scores, based on WHO child growth standards.

### Physical activity

The average ENMO from 12am one day to 12am the following day, i.e., 24h average ENMO, was 13.1 (SD = 3.7, range = 7.0–26.2) m*g*. The average ENMO between waking up and going to bed, i.e., daytime average ENMO, was 17.5 (SD = 5.1, range = 9.4–33.6) m*g* (Table 2).

**Table 2:** Time spent in different levels of PA throughout the day.

	Mean	Standard deviation	Range
Inactivity, min	862.6	66.2	690.0–1029.4
Light activity, min	88.4	22.6	44.6–136.1
Moderate activity, min	25.8	16.7	4.0–77.5
Vigorous activity, min	0.5	1.8	0.0–13.0

### Sleep

Mean sleep duration was 7.7 (SD = 1.1, range = 4.7–10.9) hours (Table 3). No patients were identified as sleeping during the day.

**Table 3:** Sleep parameters.

	Mean	Standard deviation	Range
Sleep duration, min	461.0	68.4	280.9–654.0
Sleep efficiency, 0–1	0.88	0.10	0.31–0.96
Awakenings, n	1.45	1.25	0–5.4
WASO^a^, min	54.6	35.8	17.4–192.8

^a^ Wake After Sleep Onset.

### Sleep duration and age

**Fig 1:**
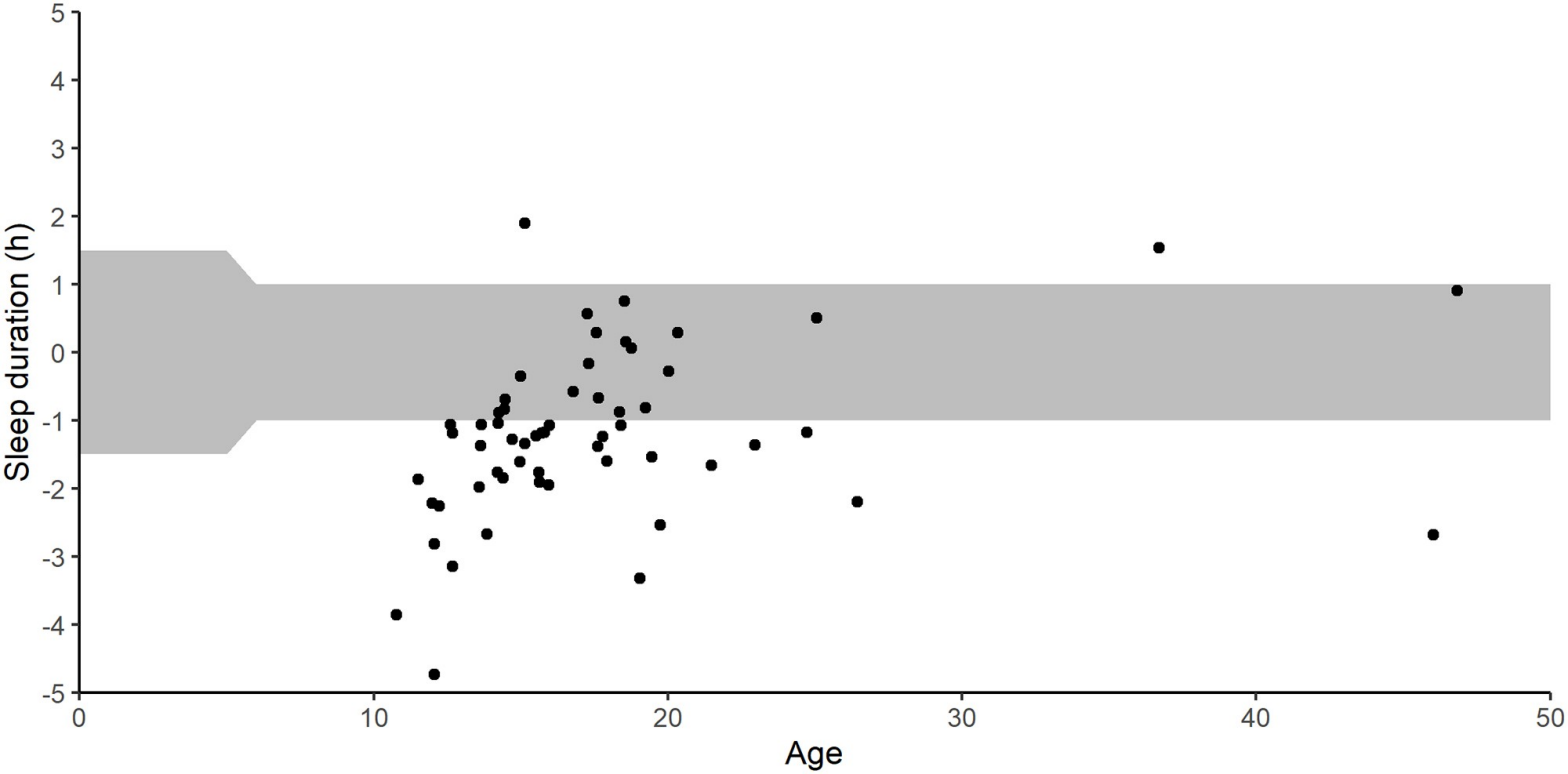
Scatter plot with deviation (in hours) from recommendations of sleep duration on the y-axis and age on the x-axis. The grey area displays the recommended interval of sleep.

There was a significant difference (p = 0.011) across groups in whether they received the recommended amount of sleep during treatment (Table 4). A higher number of school aged children and adults failed to reach recommendations compared to teens and young adults (Fig 1).

**Table 4:** Mean sleep duration, sleep duration recommendation and ratio of participants reaching recommendations for each age group.

Age groups	Sleep duration, min (SD)	Sleep duration recommendation, min *	Enough sleep, ratio (%)
School age (6–13 yrs.)	460.4 (66.9)	540–660	0/13 (0)
Teen (14–17 yrs.)	482.8 (52.2)	480–600	9/26 (35)
Young adult (18–25 yrs.)	428.6 (68.7)	420–540	8/15 (53)
Adult (26–64 yrs.)	443.5 (128.1)	420–540	1/4 (25)

* Sleep duration recommendations for age ranges were derived from the National Sleep Foundation [42].

### Demographic data vs physical activity and sleep parameters

Correlation between the demographic data (age, BMI, illness onset, and illness duration) and physical activity and sleep parameters were all low, ranging from R = <0.001 to 0.184 (Table 5).

**Table 5:** Correlation coefficients (R) between demographic data and PA and sleep parameters.

	Age	BMI	Illness onset	Illness duration
24h average ENMO	0.016	0.112	<0.001	0.033
Inactivity	<0.001	0.017	<0.001	0.002
Light activity	0.042	0.066	<0.001	0.077
Moderate activity	0.009	0.184	0.001	0.020
Vigorous activity	<0.001	0.036	<0.001	<0.001
Sleep duration	0.007	0.004	0.003	0.006
Sleep efficiency	<0.001	0.004	<0.001	<0.001
Awakenings	0.002	0.030	0.010	0.016
WASO	<0.001	<0.001	<0.001	<0.001

## Discussion

During the first week of inpatient treatment AN patients displayed low levels of physical activity. Meanwhile, few patients managed to reach sleep duration recommendations, especially in the lower age groups.

There is no standard unit for measuring PA in patients with AN and while counts seems to be the most common, differences in devices, definitions, and proprietary algorithms have resulted in a daily difference of 4971 to 230067 counts between studies [44, 45]. Hence, comparison between studies in the field are discouraged at this point. The currently used material and methods should increase result reproducibility. The main reasons for this is that the formatting and analysis method used handles raw data (m/s^2^ or gravitational acceleration) and can be used by multiple accelerometer devices (Axivity, GENEActive and ActiGraph). The largest study where they have employed similar material and methods is the UK Biobank study, where data from more than 100.000 individuals was collected [46]. Compared to the UK Biobank study, the 24h average ENMO of patients in the current study was lower than women (23.9) and men (22.9) in the least active age group (75–79 years of age) [46]. In addition, the 24h average ENMO of the current study was lower than groups know to have a low PA level, such as adults office workers (26.9), adults with diabetes type 2 (22.0) and postmenopausal women (27.1) [47]. Infact, 24h average ENMO in this study was almost as low as the activity of standing still (10.8) in a study where they investigated intensity thresholds for specific activites, which suggests the average activity level of patients during the measurement period was low [48]. Contrary to previous research, there seemed to be no subgroup with high activity levels, as the max average ENMO was only 26.2 [8, 16, 18, 19]. In the present study, estimates of minutes spent in moderate to vigorous PA (26.3 min) was less than one third of three other studies which reported these measurements in this patient group (151.9, 82 and 99.5 min) [16, 19, 49]. Meanwhile, the sedentary (inactivity) time was almost twice that of previous studies (495 and 496 min) [19, 49]. However, due to the difference in devices, algorithms, cut-off points for activity levels, outcome measures and body placement these results are difficult to compare and further investigation is required. While there is no consensus regarding PA recommendations for AN patients, both questionnaire and accelerometer based studies suggest that high levels of PA have a negative effect on treatment outcomes [22–24]. Even though the low activity displayed by patients in the current study would suggest that their treatment will not be negatively influenced by to high PA, longitudinal studies are required to determine such a relationship.

When comparing the average sleep duration to the average age, on a group level, it was only slightly below recommended levels. The sleep duration was slightly higher than what was estimated in the UK biobank study (7.3 hours) using the same algorithm [50]. The sleep duration in the current study was similar to two other studies using accelerometers in this patient group (444 and 446.5 minutes), but wake after sleep onset was higher (26 and 49 min) [32, 33]. The sleep efficiency was comparable to a study where patients had a BMI of 14.4 (0.88) [33], but lower than a study where patients had a BMI of 16.8, in which patients (0.94) had slightly better sleep quality than healthy controls (0.94) [32]. Number of awakenings has not been reported using accelerometers, but compared to polysomnographic studies the number of awakenings was much lower. A reason for this could be the criteria to only include awakenings longer than 5 minutes, where in the only polysomnography study that measured both awakenings and WASO the average awakened period was 1.5 minutes [26]. As is the case with physical activity, further investigation with reproducible methods (result reproducibility) is required to reach a conclusion on sleep in AN.

When comparing the sleep duration of each patient with the recommended sleep duration for their respective age group it became apparent that the youngest patients (and possibly the oldest) were at higher risk of not meeting the sleep recommendations. Meanwhile, young adults were at lowest risk. This finding has not been reported in previous studies and the primary cause is the increased need for sleep in younger age groups. It should be noted that the prevalence of sleep problems of otherwise healthy individuals in the younger age groups are expected to be high [51] and may have been exacerbated by having been admitted to inpatient care. Currently the treatment protocol focuses less on sleep duration and quality, compared to PA and eating behaviour. Therefore, sleep quality in treatment could likely be improved with only slight alterations to the current treatment protocol, for example, implementing different bedtimes for different age groups.

There was an expectancy to find a relationship between age of onset of disorder, disorder duration or BMI and PA and sleep, since some of these behaviours have been associated with poor treatment outcomes [24, 34]. However, the correlation between demographic variables (age of onset of disorder, duration of disorder and BMI) and PA and sleep outcome variables was low. This may be the result of the treatment protocol, which is highly supervised and where there are strict protocols to reduce PA and increase food intake. It is also possible that the relative change in PA and sleep during treatment is more important in determining treatment success. It would therefore be interesting to see the effect on PA and sleep once patients move to outpatient care and how these variations influence treatment outcomes. In one such study, researchers found no significant effect of physical activity on recovery rates [21]. Meanwhile, a more recent study showed that patients with higher light physical activity at admission had a higher inpatient BMI percentage gain, but a loss of BMI percentage gain at follow-up [52]. A reason for these discrepancies could be different patient groups or treatments, but also differences in methods (i.e., activity metrics, body placement, devices and proprietary algorithms).

One strength of the study was that an open-source algorithm was used to format and analyse the data and that raw data was accessible from the accelerometer, improving result reproducibility and transparency. Another strength was the large number of patients and high data retention rate (loss due to corrupt data and low compliance was 13%). In addition, all patients were measured at the same time in treatment (admission to inpatient care), ensuring measurements were made in a homogenous group. This resulted in a sex distribution which agrees with what is expected in this patient group and a degree of patient emaciation which seems comparable to previous studies that used objective methods to measure PA and sleep in an inpatient ward (where BMI ranged from 14.3 to 16.8) [32, 49]. However, only a few studies have been conducted on patients this young [21, 45], where most studies have been on patients with an average age above 20. This also explains the relatively short illness duration, which was similar to another study in a younger age group [45]. One weakness of the study was that only one wrist worn accelerometer was used per patient. While wrist placement seems ideal for measuring sleep, it usually provides less accurate measurements on PA than accelerometers placed on the waist or thigh. Another weakness was that no measurements were made before or after treatment, making it impossible to quantify the effect of treatment on PA and sleep. In addition, the sleep recommendation analysis for the oldest age group should be interpreted with care due to the low number of participants between 25 and 64 years of age.

In conclusion, the patients spent most of their time inactive at the beginning of treatment. Meanwhile, most patients fail to reach sleep recommendations and the average sleep efficiency is lower than expected for healthy controls. Due to the use of raw data and opensource software the current method should improve result reproducibility and enable comparison to other points in treatment of AN patients, as well as across clinics employing different treatment methods.
